# Diabetes Distress in patients of Type 2 Diabetes Mellitus at a Teaching Hospital in Pakistan

**DOI:** 10.1192/j.eurpsy.2025.1818

**Published:** 2025-08-26

**Authors:** M. Fatima

**Affiliations:** 1 Academic Department of Psychiatry and Behavioral Sciences, King Edward Medical University Lahore, Lahore, Pakistan; 2 Psychiatry, Dublin North Mental Health Service, Dublin, Ireland

## Abstract

**Introduction:**

Diabetes management in lower-middle-income countries is becoming more challenging with an ever-increasing prevalence. It encompasses emotional distress, regimen, physician-related & interpersonal distress. Diabetes Distress is associated with poor psychosocial well-being & self-management, depression, medication non-adherence, poor glycemic control, & diabetic complications. There is a shortage of literature on diabetes distress, in terms of original studies from Pakistan.

**Objectives:**

To assess the prevalence of Diabetes Distress and its associated factors in Type 2 Diabetes Mellitus (T2DM) patients attending a teaching hospital.

**Methods:**

This cross-sectional study was conducted at the Endocrinology clinic, Mayo Hospital Lahore Pakistan from November 2023 to May 2024. 254.

Type 2 Diabetes Mellitus patients, 30-80 years old, diagnosed according to American Diabetes Association diagnostic criteria at least 6 months before data collection were included. Those who had any terminal illness, substance dependence, active psychosis, or couldn’t understand & speak Urdu were excluded. Diabetes Distress Scale-17 (DDS-17) was used for data collection. It has four dimensions & 17 items, each rated on a 6-point Likert scale. SPSS 26 IBM Corp was used for data analysis. Quantitative variables were presented as Mean (SD) & qualitative as f(%). The association of diabetes distress with various factors was elicited using the Chi-square test of independence at α ≤ 0.05.

**Results:**

The mean age of the respondents was 53.62 ± 8.55 & duration of illness 8.86 ± 6.7. Mean DDS-17 scores were 2.732 ± 1.036 SD, equivalent to moderate level distress. Emotional Burden & Regimen-related distress had comparatively higher scores than Physician-related & interpersonal distress. 40.9% had high & 33.5% had moderate level distress. Only 25.6% of participants had little to no diabetes distress. There was no statistically significant relationship between diabetes distress severity & age, employment, marital status, & urban or rural living. There was a statistically significant relationship of diabetes distress severity with gender (p=0.001), education (p=0.05), socioeconomic status (p=0.01), & illness duration (p=0.05). Female gender, lack of education, and low & middle socioeconomic status had considerable association with greater severity of diabetes distress. Participants with < 1-year duration of diabetes distress had a higher proportion reporting little to no diabetes distress.

**Conclusions:**

This study concludes that a considerable proportion had moderate to high level distress: emotional burden and regimen-related distress being of the utmost significance. The concerted efforts from all stakeholders will ensure early and timely screening of Diabetes Distress to avoid adverse psychosocial and patient outcomes. Integrating mental health with diabetes care in Pakistan could be the first step towards improving patient outcomes.

**Disclosure of Interest:**

None Declared

